# Testing a Computational Model of Interruptions: The Effects of Time Pressure on Interruption and Response Decisions

**DOI:** 10.1177/00187208251388356

**Published:** 2025-10-23

**Authors:** Emma B. Knight, Hector Palada, Andrew Neal, Penelope Sanderson, Timothy Ballard

**Affiliations:** 1 1974 The University of Queensland, Australia

**Keywords:** distractions and interruptions, decision making, experimental design, computational modeling, team communication

## Abstract

**Objective:**

The objective of this study is to empirically test a computational model of interruptions processes and effects, and to compare an alternative model to determine which best explains interruption and response decision making.

**Background:**

Interruptions in safety-critical environments (e.g., hospitals) can lead to an increased risk of error for the person being interrupted (the interruptee) but may be necessary for the person doing the interrupting (the interrupter) to maintain safety. Little research has considered the perspective of both the interrupter and interruptee.

**Method:**

We tested a computational model of interruption and response decision processes through an experiment where participants (*n* = 312) worked as a nurse in a simulated clinical team. We examined how task progress, time remaining, and time pressure influenced decisions and compared the model with an alternative that allowed the effects of time pressure to be non-monotonic.

**Results:**

Using Bayesian hierarchical modeling, we found that a non-monotonic model best explained interruption decisions. Participants were biased toward interrupting, with time pressure only influencing decisions when it was very high. Contrastingly, the monotonic model best explained response decisions. Participants were more likely to accept interruptions as the interrupter’s time pressure increased in comparison to their own.

**Conclusion:**

Time pressure has a non-monotonic influence on interruption decisions, but a monotonic influence on response decisions.

**Application:**

Findings can inform interventions to consider the interruptions process from the perspective of both the interrupter and interruptee. Interventions could focus on training workers to more accurately assess time pressure when making interruption decisions.

Interruptions occur frequently in safety-critical work environments, such as healthcare (Janssens et al., 2024; Westbrook et al., 2010, 2018), military (Loft et al., 2015), and aviation environments (Alqahtani, 2014; Gontar et al., 2017; Latorella, 1998; Wilson et al., 2020) where team members rely on help from one another to complete tasks efficiently and safely. Interruptions can be problematic for the team member that is interrupted—the interruptee—as they must put their task on hold and may experience an increased risk of error when they resume their task (Bailey & Konstan, 2006; Chen et al., 2022; Grundgeiger & Sanderson, 2009; Johnson et al., 2018; Loft et al., 2015; Santomauro et al., 2021; Westbrook et al., 2010; Wu et al., 2023). Interventions to reduce interruption costs have been developed, including nurses in hospitals wearing “do not disturb” vests or working in “interruption free” zones (Dall’Oglio et al., 2017; Huckels-Baumgart et al., 2017; Raban & Westbrook, 2014; Verweij et al., 2014).

However, such interventions have not been widely successful (Gao et al., 2017; Ratwani et al., 2014; Westbrook et al., 2017). Many interventions have focused on reducing interruptions for the interruptee, with little consideration of the necessity of interruptions for the team member doing the interrupting—the interrupter—to maintain safety (Gao et al., 2017). For example, in a hospital, a nurse may interrupt to seek help with a critical patient or to confirm a medication dosage (Coiera & Tombs, 1998; Spencer et al., 2004). There has been little research that examines interruptions at a team or system level—considering the perspective of both the interrupter and interruptee and the emergent effect of interruptions on team performance (McCurdie et al., 2017). Such complexities can be difficult to capture with verbal theories, which are predominantly used in this literature (Grundgeiger et al., 2010; Westbrook et al., 2010). To capture the effects of interruptions at a team level, Knight et al. (2023) developed a computational model based on the interruptions literature (available at https://osf.io/asgp7/). The model represents how team members make decisions to interrupt and respond to interruptions, by considering factors such as time pressure, while completing a series of tasks. However, the predictions of the model have not been tested empirically.

In this study, we test a computational model of interruptions with an experiment where team members complete tasks in a time-pressured environment while making decisions to interrupt and respond to interruptions from one another. We use Bayesian parameter estimation to test Knight et al.’s (2023) model and compare alternatives, to see which best accounts for participants’ decisions to make or accept an interruption, and to derive insights about their decision processes. Findings can provide a better understanding of how interruptions occur between team members and can guide the development of interventions that are better suited for safety-critical teams.

## Interruption and Response Decisions

While completing tasks, team members may require help from one another. For example, in a hospital ward, a second nurse may need to double-check a medication dosage. In an aircraft cockpit, a pilot may need to alert another pilot to an abnormal event. Studies suggest that when team members in safety-critical environments need to interrupt, they compare the benefit of interrupting with the potential cost to the interruptee (Colligan & Bass, 2012; Knight, Sanderson, et al., 2023). Likewise, if a team member is interrupted, they assess the benefit and cost to determine whether to engage in the interrupting task. The perceived benefit of an interruption is influenced by urgency, which is a function of the time pressure under which the interrupter is working (Gao, 2018) and the importance of the interruption for maintaining productivity and safety (Moody-Williams, 2020). Factors that can contribute to the cost of an interruption include the timing of the interruption—whether an interruption occurs in between tasks or in the middle of one (Altmann & Trafton, 2002; Bailey & Konstan, 2006; Bala, 2012; Grundgeiger et al., 2010) and whether it is socially appropriate for the interruptee to handle an interruption (Rivera, 2014). Research suggests that team members also consider the length of the interruption, as longer interruptions may lead to an increased resumption time (i.e., time taken to resume the primary task) and cause the interruptee to fall behind on work (Ebright et al., 2003; Hasegawa et al., 2021; Monk et al., 2008). Interruptions can be more costly if they are cognitively similar to the primary task (e.g., both medication calculations) as the interruptee may confuse the two tasks (Bala, 2012; Lee & Duffy, 2015; Trafton & Monk, 2008). If the interruptee can maintain visual cues for their primary task (e.g., their patient’s chart) while completing the interruption, this can also help task resumption, making the interruption less costly (Dismukes, 2006; Grundgeiger et al., 2010). Finally, interruptee cost can be influenced by urgency, specifically the time pressure under which the interruptee is working (Ebright et al., 2003) and the potential risk to safety if a team member is interrupted (Klemets & Evjemo, 2014).

Time pressure is particularly important in both interruption and response decisions as team members in safety-critical environments often work under tight deadlines. For example, air traffic controllers may need to promptly modify flightpaths before two conflicting aircraft breach separation. Similarly, when discharging patients from an ICU to a general ward, nurses try to complete the process quickly to free up beds for incoming patients (College of Intensive Care Medicine of Australia and New Zealand, 2011; Lin et al., 2009). However, there may be situations when teams have more time and fewer tasks to complete so experience less time pressure, or when individuals operate under different levels of time pressure. Team members may be more likely to interrupt or accept an interruption if the time pressure of the interrupter is greater than that of the interruptee (Ebright et al., 2003; Rivera, 2014). Some team members may also be more inclined to interrupt or accept interruptions than others. A team member’s bias toward interrupting or accepting may be influenced by their level of experience with the team and interruptions (Abdelhadi et al., 2022; Rivera, 2014).

Research suggests that once the above factors have been assessed, the interrupter decides whether to interrupt. If they do, the interruptee decides how to respond. Interruptions can be problematic and frustrating for the interruptee, as they must pause their primary task and risk falling behind (Grundgeiger & Sanderson, 2009; Janssens et al., 2024; Santomauro et al., 2021; Westbrook et al., 2010). When they resume their primary task, the interruptee may experience an increased resumption lag and an increased risk of error, meaning they require time to resolve errors (Grundgeiger et al., 2010; Santomauro et al., 2021; Weigl, Müller, et al., 2012; Westbrook et al., 2010). However, interruptions can help the interrupter to progress with their tasks and maintain safety (Berg et al., 2013; Gao et al., 2017; McCurdie et al., 2017; Weigl, Hornung, et al., 2012). It is important to consider both the perspective of the interrupter and the interruptee when studying interruption processes and effects.

## A Computational Model of Interruptions

Knight et al. (2023) developed a computational model to better understand the complex dynamics involved in interruptions. Knight et al.’s (2023) model was developed to represent two team members working on tasks to complete two goals each, under a deadline. The model represents team members making decisions to interrupt and respond to interruptions from one another and captures the effects of such decisions, including any post-interruption errors. Knight et al. (2023) applied the model to the context of a hospital ward, where team members are nurses, but the model can be used to represent any context where members complete tasks and interact through interruptions.

The model assumes that when deciding to interrupt or respond to interruptions, the interrupter and interruptee weighs up the costs and benefits of the interruption. However, the interrupter and interruptee can vary in their bias toward interrupting or accepting interruptions and their sensitivity to the costs and benefits. The probability of team member *i* deciding to interrupt another team member at time *t* is calculated as follows:

(1) $${p\left(interrupt\right)}_{it}=\frac{1}{e^{-\left[\alpha_{i}+\beta_{i}\times \left(b_{it}-c_{it}\right)\right]}}$$

Likewise, the probability of team member *i* deciding to accept an interrupt at time *t* is calculated as follows:

(2) $${p\left(accept\right)}_{it}=\frac{1}{e^{-\left[\gamma_{i}+\delta_{i}\times \left(b_{it}-c_{it}\right)\right]}}$$

In both equation (1) and equation (2), *b* represents interrupter benefit and *c* represents interruptee cost. The α parameter represents interrupter bias and the γ represents interruptee bias. A bias greater than zero indicates a tendency to interrupt, or accept interruptions, and a bias less than zero indicates a tendency to not interrupt, or to not accept interruptions. The β parameter represents interrupter sensitivity and the δ parameter represents interruptee sensitivity—the extent to which they are each influenced by the benefit–cost difference when making interruption or response decisions. For example, people would be more likely to interrupt, or accept interruptions, if the benefit of the interruption was greater than the cost, particularly if they were sensitive to that difference or if their bias toward interrupting or accepting interruptions was high. The bias (α, γ) and sensitivity (β, δ) are free parameters which can be estimated from data.

In both equations, interrupter benefit (*b*) and interruptee cost (c) capture the time pressure and importance of the interrupting task and interruptee’s tasks respectively. A team member’s time pressure reflects the demand to complete tasks within a given deadline. Time pressure ( ${TP}_{it}$ ) is calculated as the ratio of the time required (*TR*) for the team member to complete all outstanding tasks, to the time available *(TA)* to complete those tasks:

(3) $${TP}_{it}=\frac{TR}{TA}$$

A value greater than one indicates that the time required to complete all outstanding tasks exceeds the time available. In the current study, the importance of tasks is held constant. The model therefore assumes that the benefit of an interruption ( $b_{it}$ ) is equal to the time pressure experienced by the interrupter at time *t*, whereas the cost of an interruption ( $c_{it}$ ) is equal to the time pressure experienced by the interruptee at time *t.*

**Figure 1.**
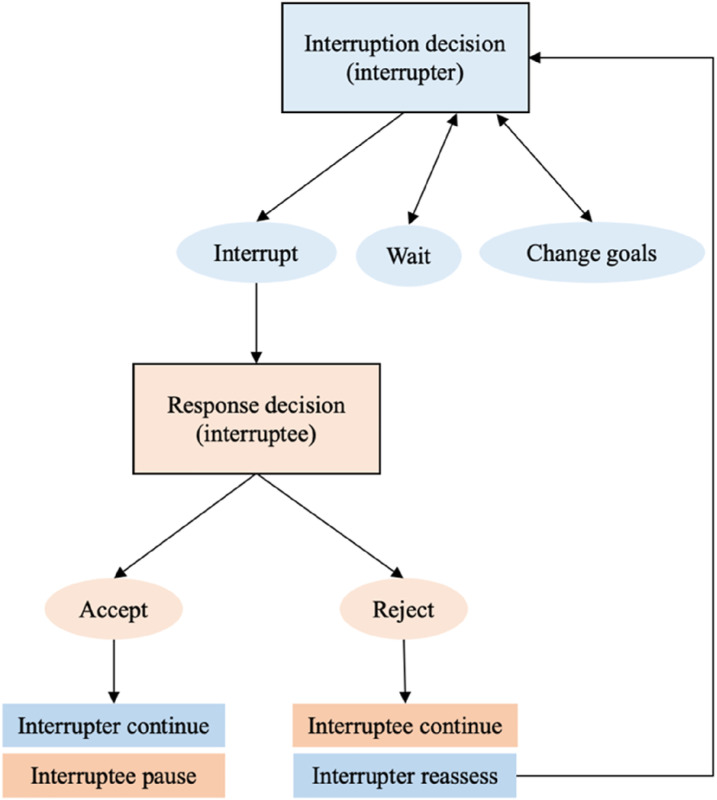
Outcomes of interruption and response decisions in the computational model. *Note.* Blue corresponds to the interrupter and orange corresponds to the interruptee.

Figure 1 shows the outcomes of different interruption and response decisions in the model. If a team member interrupts, and the interruptee accepts the interruption, the interruptee pauses their task while the interrupter progresses, having received the help they needed. However, the interruptee experiences an increased risk of error after handling the interruption. The consequences of an error depend on whether the error is detected or not. If undetected, errors have the potential to lead to harm. However, the extent to which harm occurs is highly contingent upon the type of error and the circumstances under which it is made. Studies using the Threat and Error Management model often show that errors are far more likely to be detected than undetected (Tzanetos et al., 2020; Zhao et al., 2017). The consequences of errors are therefore often reflected through the time it takes to resolve them. In the model, errors are assumed to be detected and are defined as clinical or procedural failures. If a team member makes an error, they lose progress on a task, representing time spent correcting the error (we return to the issue of undetected errors in the discussion). The amount of progress lost is determined by a variable referred to as *error impact*. On the other hand, if the interrupter decides not to interrupt, or the interruptee rejects their interruption, the interruptee continues making progress on their task, while the interrupter does not. The interrupter may switch their attention to another goal or wait to interrupt again.

## The Current Study

The aim of the current study is to test the computational model of interruptions through an experiment in which participants work on tasks in a time-pressured environment while making decisions to interrupt or respond to interruptions from one another. Specifically, we use an experimental task that simulates nurses working to discharge patients from an ICU, while interacting through interruptions. The process of patient discharge from an ICU was chosen to represent a variety of nursing tasks in a safety-critical workplace where team members often work under time pressure and where interruptions are common and can have severe consequences (Alvarez & Coiera, 2005; Crocker & Keller, 2005; Khairat et al., 2021; Westbrook et al., 2010, 2018).

While the experimental setting is an ICU, the computational model is a general model of interruption processes and effects, so the results regarding how people make decisions to interrupt and respond to interruptions can generalize to other safety-critical contexts where interruptions occur. We chose to assess the impacts of two factors on interruption and response decisions. The first was time pressure, as this is a key driver of these decisions across a range of safety-critical environments. We manipulated time pressure by varying the deadline to which participants were aiming to discharge patients. Second, we manipulated error impact, to represent the fact that potential errors made by an interruptee after an interruption could take more or less time to resolve and therefore affect the progress of the interruptee. We explore other potentially influential factors in the discussion.

To understand decision processes, we first test how progress on tasks and time remaining to complete tasks affects interruption and response decisions. Next, we fit the interruption decision and response decision models to experimental data to assess how accurately the model predictions align with observed decisions. Using Bayesian hierarchical modeling, we estimate bias and sensitivity in the model to determine the extent to which participants tend to interrupt or accept interruptions, and how sensitive they are to differences between interrupter benefit and interruptee cost. Finally, we compare the hypothesized monotonic model to a plausible alternative model that assumes the effects of time pressure on the cost and benefit of an interruption are non-monotonic.

## Method

### Participants

Participants (*n* = 312, 240 females, 72 males) were undergraduate psychology students (*n* = 222) and members of the paid research participation pool (*n* = 90) at The University of Queensland, with an age range of 17–57 years (*M* = 21.18, *SD* = 4.99).

### Experimental Paradigm

**Figure 2.**
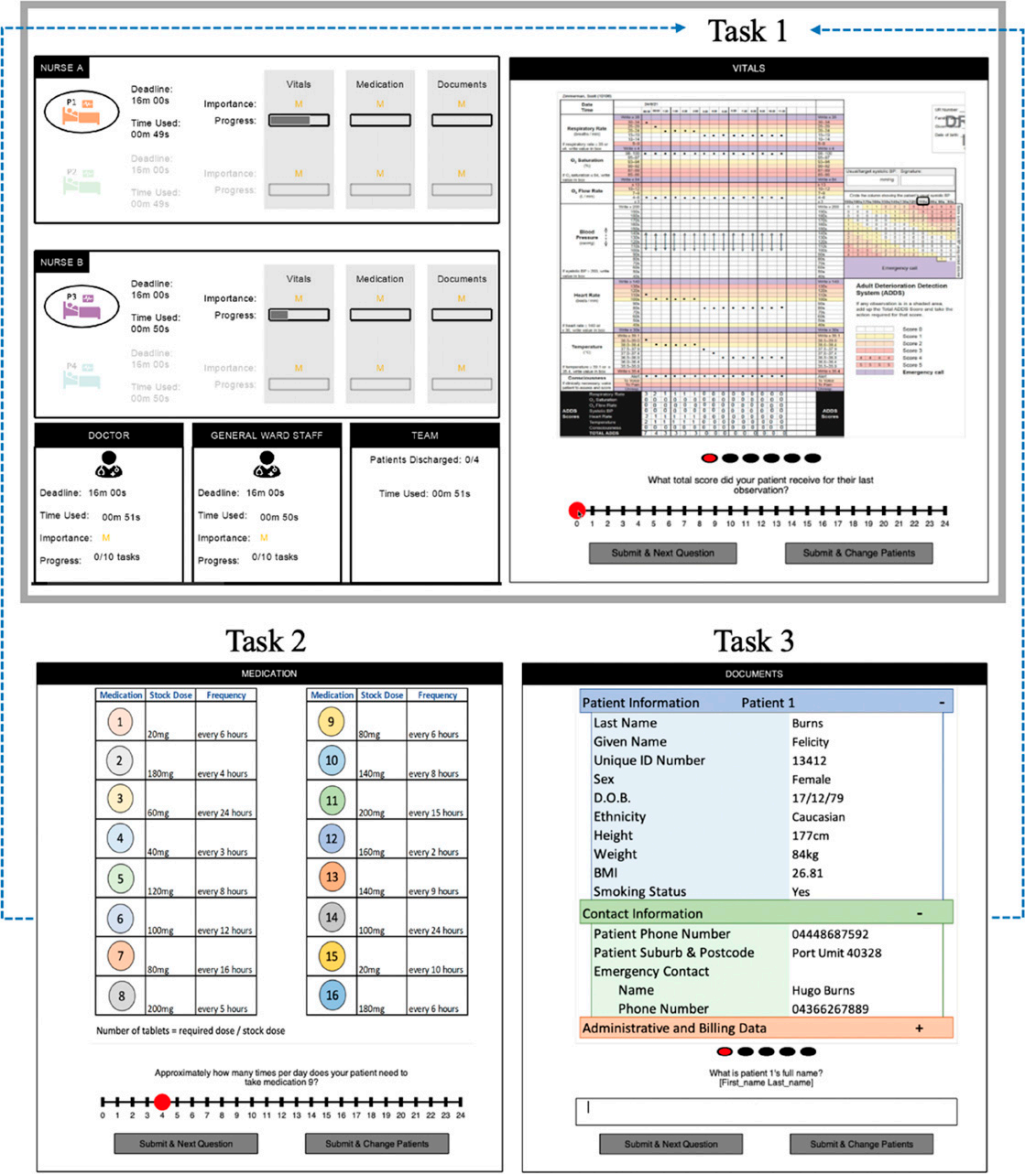
Experiment interface displaying the progress of all clinicians (top left) and the three tasks (top right, bottom left and right). *Note.* Participants were shown the interface outlined on the top, with the three tasks completed on the right side of the interface (as indicated by the blue arrows).

The experimental paradigm is shown in Figure 2. Participants acted as one of two nurses, while the other nurse, the doctor, and general ward staff were computer simulated. Each nurse managed two patients: P1 and P2 for the participant and P3 and P4 for the simulated nurse. The patient that each nurse was working on was circled. Nurses completed three consecutive tasks per patient—checking vital signs, preparing medication, and completing discharge documentation—to discharge them from the ICU to a general ward. Nurses’ progress on tasks was captured through progress bars, along with the importance of the task represented as low (*L*), medium (*M*), or high (*H*). Participants could also see how much time within which they, and the other clinicians, were aiming to complete all tasks (*Deadline*) and how much time had elapsed (*Time Used*). In the Team box, they could see how well the team was progressing. The experimental block was complete once the team had completed all tasks. The experiment code and supporting materials, including training, are available on the Open Science Framework (https://osf.io/y9jba/).

Two Australian nurses with experience working in ICUs were consulted to design the experiment and pilot testing was conducted with 39 participants. Participants accuracy (*M* = 0.60, *SD* = 0.49), question response time in seconds (*M* = 11.20, *SD* = 13.05), frequency of deciding to interrupt (68%), and frequency of deciding to accept interruptions (59%) was measured to ensure the tasks were sufficiently challenging, to inform the deadlines in the experiment, and to ensure that different interruption and response decisions were being made.

### Tasks

Participants completed three tasks for each patient shown on the right of the interface (Figure 2; labeled as Vitals, Medications, and Documents). Task represented activities that are completed during patient discharge from an ICU (Crocker & Keller, 2005). The simulated nurse progressed on tasks at a rate consistent with the average performance in the pilot study, with a response time of 11 s per question, and a 5% rate of error. Participants started working on Patient 1 but could switch between patients throughout.

In the first task—checking vital signs—participants were shown a Queensland Adult Deterioration Detection System (QADDS) vitals chart (Australian Commission on Safety and Quality in Healthcare, 2018; Dwyer et al., 2019) with observations of the simulated patient’s vital signs (e.g., heart rate, respiratory rate, and temperature) over time. Participants were required to look to the chart to answer 13 questions such as *“What total score did your patient receive for their last observation?”*. Participants responded by selecting the number that aligned with the observation score.

The second task—medication preparation—showed a chart of medications, their stock dosage and required frequency of use. Participants answered 16 questions such as *“Approximately how many times per day does your patient need to take medication 9?”,* by selecting the appropriate number.

In the third task—discharge documentation—participants could switch between charts that documented general and medical information about both patients (e.g., age, ward number, and diagnosis). Participants looked to the charts to complete documentation by typing answers to 24 questions such as *“What is Patient 1’s full name?”*.

If participants got a question wrong, they were required to wait a specified number of seconds before attempting the question again. The wait time was experimentally manipulated and represents the time taken to resolve an error. They were given three attempts at answering a question before being moved to the next question.

#### Interruption and Response Decisions

##### Deciding to Interrupt

**Figure 3.**
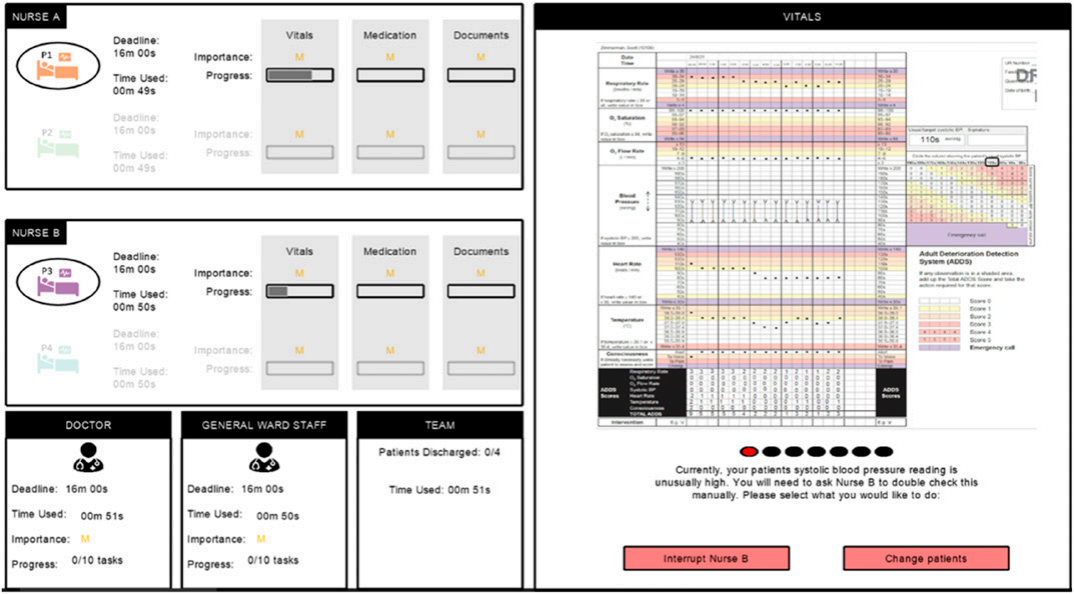
Decision to interrupt in the experiment.

At certain points throughout tasks, participants were prompted to make interruption decisions, with the timing of prompts varying across tasks. For example, while checking their patient’s vital signs, participants needed the simulated nurse to check a blood pressure reading (Figure 3). They were given two options: *Interrupt* or *Change Patients*. Participants could see the progress of themselves and the simulated nurse, as well as their deadlines and current time used when making the decision.

The outcomes of interruption decisions were the same as in the model (Figure 1). Participants could choose to change patients (i.e., goals), but if they needed to interrupt for both patients, they could not progress until they decided to interrupt. If participants chose to interrupt, the response of the simulated nurse was determined using the response decision equation from the model (equation (2)), where interrupter benefit and interruptee cost were both determined by time pressure and importance for patient safety. Time pressure was calculated using the equation from the model (equation (3)), as the ratio of time required to complete all remaining tasks (based on the time of 11 s per task) to the time available to reach the deadline. Importance was represented for each task as L (1), M (5), or H (10). Interruptee bias and interruptee sensitivity were equal to 0 and 1 respectively, so only the difference in benefit and cost between the interrupter (participant) and interruptee (simulated nurse) influenced the simulated nurse’s response decisions. If the simulated nurse accepted an interruption, they paused their own task while the participant progressed. After responding to an interruption, the simulated nurse experienced an increased risk of error for the next eight questions. If they made an error post-interruption, their time used increased, representing time spent resolving an error. If the simulated nurse rejected the interruption, they continued making progress, while the participant had to change patients or continue interrupting before they could progress.

##### Responding to Interruptions

**Figure 4.**
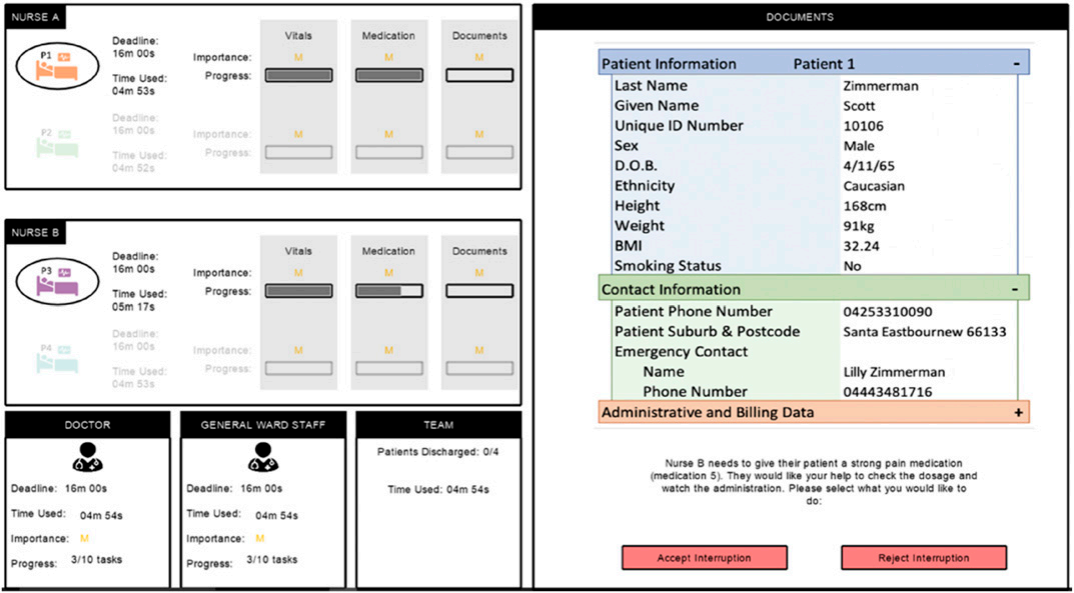
Responding to interruptions in the experiment.

Throughout tasks, the simulated nurse was occasionally required to interrupt the participant (Figure 4) using the equation from the model (equation (1)), with the timing of interruptions varying across tasks and blocks. If the simulated nurse interrupted, the participant was shown a question from the simulated nurse, asking them to check a medication dosage, for example. They could choose to *Accept Interruption* or *Reject Interruption*. If they rejected the interruption, or the simulated nurse decided not to interrupt, the participant could progress on their task (Figure 1). However, the simulated nurse would need their interruption accepted to progress on that task, so would either change patients or continue to interrupt every 11 s in future until the participant accepted. If the participant accepted the interruption, they answered questions relating to the simulated nurse’s patient (with the simulated nurse’s progress increasing) before continuing their own task. If participants made an error post-interruption, they were required to wait a specified number of seconds before attempting the question again.

### Manipulations

We employed a 3 (*deadline*: 960, 1320, 1680 s) × 2 (*error impact*: 3, 6 s) mixed experimental design. Deadline was manipulated between subjects and error impact was manipulated within subjects. Task importance was constant at a medium level (5) for all clinicians and patients. Participants completed two experimental blocks, where they were randomly assigned a deadline across both blocks, and assigned a different, counterbalanced error impact in each block.

#### Deadline

Deadline represents the time in which the discharge process should be completed for all patients. Deadline was randomly assigned to the participant and simulated nurse’s patients as either 960, 1320, or 1680 s. Participants could see the deadline and time used for themselves and the simulated nurse (Figure 2), to assess their progress. Deadline was determined via pilot testing, where the medium deadline (1320 s) was pilot participants mean completion time. The short and long deadlines were 360 s shorter and longer. Once participants had less than 120 s remaining before the deadline, the time used flashed red, to indicate time pressure.

#### Error Impact

Error impact was represented as the time required to resolve an error, counterbalanced to be 3 or 6 s in each of the two blocks. If participants made an error, a message popped up stating “Incorrect. Please wait X seconds,” with X set to 3 or 6 s. Participants needed to wait the specified time before they could continue with their task. If the simulated nurse made an error, their *time used* increased by 3 or 6 s.

### Procedure

Participants first completed 20 min of instructions and practice questions using Qualtrics survey software. Participants were instructed that they would be making interruption and response decisions and could look to the progress bars, deadline, and time used of themselves and the simulated nurse. The consequences of interruption and response decisions were also explained to them. Participants then completed two blocks of the experiment, with each block consisting of three tasks for each of two patients. This research complied with the American Psychological Association Code of Ethics and was approved by the Institutional Review Board at The University of Queensland. Informed consent was obtained from each participant.

## Results

We first report descriptive statistics from the experiment. Next, we examine how progress on tasks and time remaining to complete tasks affects participants’ likelihood of interrupting and likelihood of accepting interruptions. Finally, we use Bayesian hierarchical modeling to assess how accurately the underlying interruption and response decision models explain the experimental data and compare a plausible alternative model that assumes the effects of time pressure on the cost and benefit of an interruption are non-monotonic. The code for analyses and model fitting are available on the Open Science Framework (https://osf.io/y9jba/).

### Descriptive Statistics

On average, participants took 11.20 s (*SD* = 13.05) to answer questions with 75% accuracy (*SD* = 0.43). When prompted with interruption and response decisions, participants chose to interrupt 88% of the time (*SD* = 0.33) and accepted interruptions 72% of the time (*SD* = 0.45).

### The Effects of Progress and Time Remaining on Likelihood of Interrupting and Accepting Interruptions

We ran Bayesian regression models to examine how the participant’s and their coworker’s progress on tasks and time remaining to complete tasks affects the likelihood of interrupting and the likelihood of accepting interruptions (Table 1). Progress on tasks for both patients is captured by *Participant Progress* and *Coworker Progress (i.e., progress of the simulated nurse)*. Instances where the deadline had been surpassed (7% of interruption decisions and 4% of response decisions), were removed from the dataset as in these cases time pressure is undefined.

**Table 1.** Bayesian Logistic Regression Modeling Results for the Effects of Progress and Time Remaining on Interruption and Response Decisions

Outcome	Predictor	Estimate	Est. Error	95% CI
Interruption decision	Intercept	2.83	0.15	2.56, 3.12
	Participant progress	0.29	0.13	0.02, 0.55^a^
	Coworker progress	0.09	0.19	−0.27, 0.47
	Time remaining	0.02	0.14	−0.26, 0.30
	Participant progress × coworker progress	0.09	0.09	−0.09, 0.27
	Participant progress × time remaining	−0.25	0.12	−0.48, −0.02^a^
	Coworker progress × time remaining	0.30	0.11	0.09, 0.52^a^
	Participant progress × coworker progress × time remaining	0.01	0.07	−0.12, 0.15
Response decision	Intercept	1.04	0.10	0.84, 1.25
	Participant progress	1.66	0.18	1.33, 2.02^a^
	Coworker progress	−1.56	0.18	−1.91, −1.23^a^
	Time remaining	−0.08	0.12	−0.33, 0.16
	Participant progress × coworker progress	0.34	0.10	0.14, 0.53^a^
	Participant progress × time remaining	0.36	0.16	0.04, 0.68^a^
	Coworker progress × time remaining	−0.21	0.14	−0.49, 0.06
	Participant progress × coworker progress × time remaining	0.15	0.09	−0.02, 0.31

^a^Credible effect. One incomplete block was removed from the dataset when running the model with total time as the outcome.

**Figure 5.**
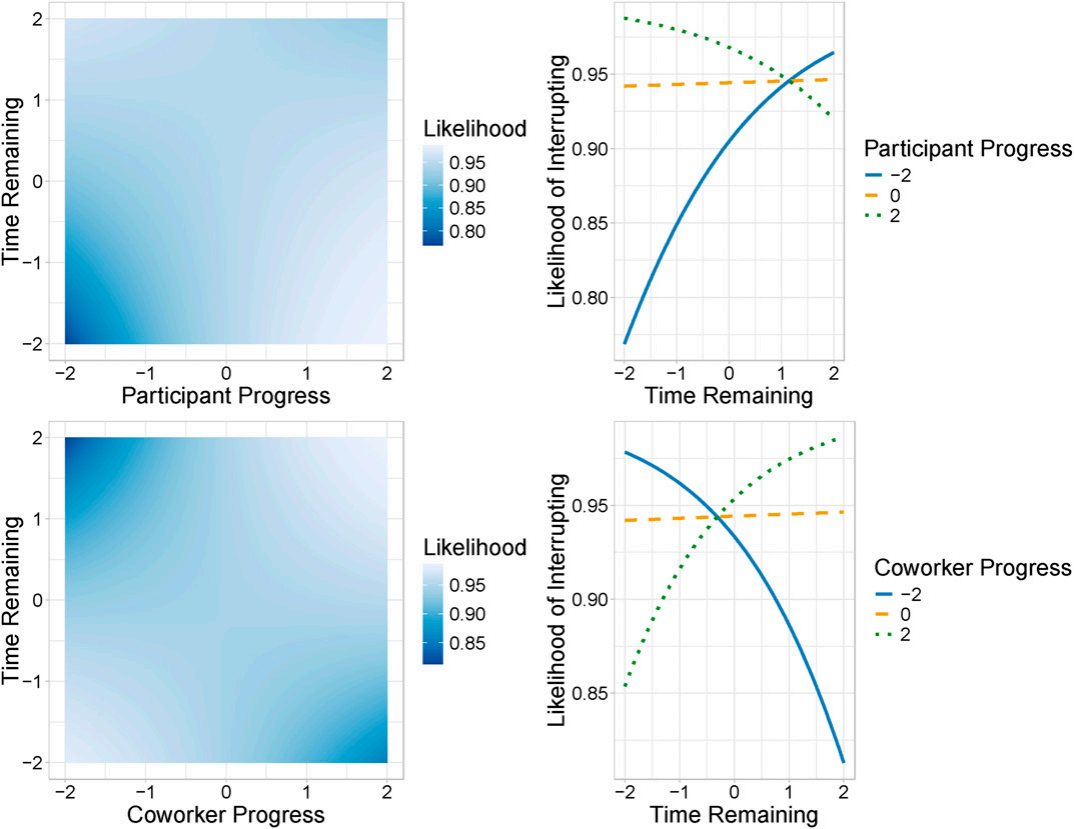
Interaction effect of participant progress and time remaining (top row) and coworker progress and time remaining (bottom row) on likelihood of interrupting. *Note.* Lighter colors on the heat maps represent a higher likelihood of interrupting. Variables were scaled with a mean of 0 and standard deviation of 1, and we plot the range from low (−2) to high (2). Time remaining was originally recorded in seconds, and progress was originally recorded as the total number of task questions completed. Likelihood of interrupting was computed through a Bayesian regression model, by modeling the probability of the binary outcomes (interrupting or not interrupting) as a function of the predictors: time remaining, participant progress, and coworker progress.

**Figure 6.**
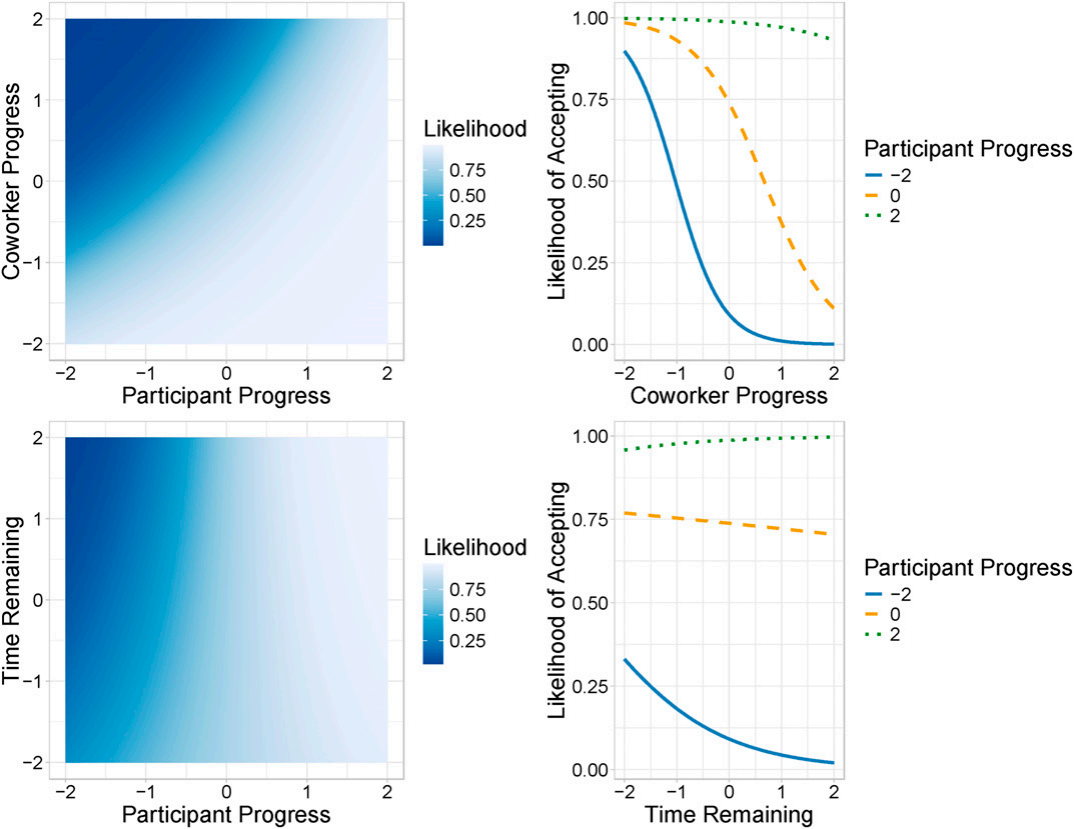
Interaction effects of participant progress and coworker progress (top row) and participant progress and time remaining (bottom row) on likelihood of accepting interruptions. *Note.* Lighter colors on the heat maps represent a higher likelihood of accepting interruptions. Variables were scaled with a mean of 0 and standard deviation of 1, and we plot the range from −2 to 2. Time remaining was originally recorded in seconds, and progress was originally recorded as the total number of task questions completed. Likelihood of accepting was computed through a Bayesian regression model, by modeling the probability of the binary outcomes (accepting or not accepting) as a function of the predictors: time remaining, participant progress, and coworker progress.

Figures 5 and 6 show the effects of standardized time remaining, participant progress, and coworker progress variables (all represented by standard deviations, ranging from −2 to 2). We found a credible main effect of participant progress on likelihood of interrupting. As participants made more progress through their tasks, they were more likely to interrupt. We also found credible interaction effects of participant progress and time remaining, and coworker progress and time remaining, on likelihood of interrupting. As shown on the top of Figure 5, when progress was high, participants were more likely to interrupt with less time remaining. However, when progress was low, participants were more likely to interrupt with more time remaining. As seen on the bottom of Figure 5, participants were also more likely to interrupt when time remaining was low and their coworker had made less progress and when time remaining was high and their coworker had made more progress. Participants were least likely to interrupt when time remaining was high and coworker progress was very low.

We found credible main effects of participant progress and coworker progress on likelihood of accepting interruptions. Participants were more likely to accept interruptions as they made more progress and were less likely to accept interruptions as their coworker made more progress. We also found a credible interaction effect of participant progress and coworker progress on likelihood of accepting interruptions (top of Figure 6). Participants were more likely to accept interruptions when their progress was greater than their coworker’s. However, when participants’ progress was high, they were very likely to accept interruptions regardless of their coworker’s progress. Further, we found a credible interaction effect of participant progress and time remaining on likelihood of accepting interruptions (bottom of Figure 6). Participants were more likely to accept interruptions when they had made more progress, at any level of time remaining, but if they had made less progress, they were more likely to accept interruptions when there was less time remaining.

### Model Fitting and Comparison: Interruption and Response Decisions

To better understand participants interruption and response decisions, we fit the interruption decision (equation (1)) and response decision (equation (2)) hypothesized models to experimental data, with interrupter time pressure contributing to interrupter benefit, and interruptee time pressure contributing to interruptee cost. Equations (1) and (2) predict a monotonic relationship, such that the difference between benefit and cost will always have an increasing relationship on interruption and response decisions. We therefore also developed and fit an alternative non-monotonic model that assumes an inverted-u-shaped relationship between benefit and cost difference on interruption and response decisions, informed by the findings that progress and time remaining have non-monotonic interaction effects on interruption and response decisions (Figures 5 and 6). The alternative model for interruption and response decisions allows the influence of time pressure on the cost and benefit of interrupting or accepting an interruption to be non-monotonic. The models require an initial calculation of the non-monotonic interrupter benefit (equations (4) and (7)) and the non-monotonic interruptee cost (equations (5) and (8)), which feed into the interruption (equation (6)) and response decision models (equation (9)).

The alternative non-monotonic model for interruption decisions is as follows:

(4) $${\;b}_{it}=\left(\tau_{i}\times {TP}_{Pt}\right)+\left(\omega_{i}\times {TP}_{Pt}^{2}\right)$$

(5) $${\;c}_{it}=\left(\tau_{i}\times {TP}_{Ct}\right)+\left(\omega_{i}\times {TP}_{Ct}^{2}\right)$$

(6) $${p\left(interrupt\right)}_{it}=\frac{1}{e^{-\left[\alpha_{i}+\left(b_{it}-c_{it}\right)\right]}}$$

Interrupter benefit (*b*_*it*_) captures the interrupter’s (i.e., participant’s) sensitivity to their own time pressure (*TP*), where subscript *p* represents the participant. Interruptee cost (*c*_*it*_) captures the interrupter’s sensitivity to the interruptee’s (i.e., coworkers) time pressure (*TP*), where subscript *c* represents coworker. Interrupter sensitivity in the assessment of benefit and cost is captured by τ (the monotonic effect) and ω (the quadratic effect). The interrupter’s bias toward interrupting is represented by α.

Next, the alternative non-monotonic model for response decisions is as follows:

(7) $${\;b}_{it}=\left(\kappa_{i}\times {TP}_{Ct}\right)+\left(\lambda_{i}\times {TP}_{Ct}^{2}\right)$$

(8) $${\;c}_{it}=\left(\kappa_{i}\times {TP}_{Pt}\right)+\left(\lambda_{i}\times {TP}_{Pt}^{2}\right)$$

(9) $${p\left(accept\right)}_{it}=\frac{1}{e^{-\left[\gamma_{i}+\left(b_{it}-c_{it}\right)\right]}}$$

Interrupter benefit (*b*_*it*_) captures the interruptee’s (i.e., participants) sensitivity to the interrupter’s (i.e., coworkers) time pressure (*TP*), where subscript *c* represents coworker. Interruptee cost (*c*_*it*_) captures the interruptee’s sensitivity to their own time pressure (*TP*), where subscript *p* represents the participant. Interruptee sensitivity is captured by κ (the monotonic effect) and λ (the quadratic effect). γ represents the interruptee’s bias toward accepting interruptions. In both models, time pressure was calculated as in Equation 3, as the ratio of the number of tasks remaining across both patients to the time available given the deadline.

We fit the hypothesized monotonic and alternative non-monotonic models using Stan (via the RStan package; Stan Development Team, 2023) and used Bayesian Hierarchical modeling to estimate model parameters (see Knight, Neal, et al. (2023) for a tutorial on these methods). We assigned broad, weakly informative priors to all population-level parameters, meaning we did not have strong prior assumptions about the likely values of parameters (see https://osf.io/y9jba/ for a breakdown of the priors and distributions).

Models were compared using the Leave-One-Out Information Criterion (LOO-IC), where lower values indicate a better trade-off between model fit and complexity, and hence a more parsimonious model (Myung & Pitt, 1997; Vandekerckhove et al., 2015). Parameter estimates for the selected models of interruption decisions and response decisions are shown in Table 2 and we use Bayesian 95% credible intervals to make inferences about model parameters. Effects are considered credible if the interval excludes zero.

**Table 2.** Parameter Estimates for the Best-Fitting Interruption and Response Decision Models

Decision	Selected Model	Parameter	M	SE Mean	SD	95% CI
Interruption decision	Non-monotonic model (alternative)	Interrupter bias (α) M	2.76	0.00	0.13	2.51, 3.03
		Interrupter bias (α) SD	0.26	0.00	0.02	0.22, 0.32
		Interrupter sensitivity a (τ) M	−0.26	0.01	0.26	−0.77, 0.29
		Interrupter sensitivity a (τ) SD	0.14	0.00	0.08	0.01, 0.30
		Interrupter sensitivity b (ω) M	0.20	0.01	0.18	−0.003, 0.69
		Interrupter sensitivity b (ω) SD	0.04	0.00	0.03	0.005, 0.12
Response decision	Monotonic model (hypothesized)	Interruptee bias (α) M	1.11	0.00	0.08	0.95, 1.27
		Interruptee bias (α) SD	0.17	0.00	0.02	0.14, 0.21
		Interruptee sensitivity (δ) M	1.37	0.02	0.41	0.50, 2.10
		Interruptee sensitivity (δ) SD	2.83	0.01	0.30	2.30, 3.45

For interruption decisions, the non-monotonic model provided the best explanation of the data (LOO-IC estimate = 2433.94, SE = 74.50), compared to the monotonic model (LOO-IC estimate = 2462.20, SE = 75.59). At the population level, only interrupter bias (α) had a credible impact on response decisions. For response decisions, the monotonic model produced the best explanation of the data (LOO-IC estimate = 2487.73, SE = 46.37), compared to the non-monotonic model (LOO-IC estimate = 2536.64, SE = 46.69). At the population level, interruptee bias (α) and interruptee sensitivity (δ) both had credible effects on response decisions.

**Figure 7.**
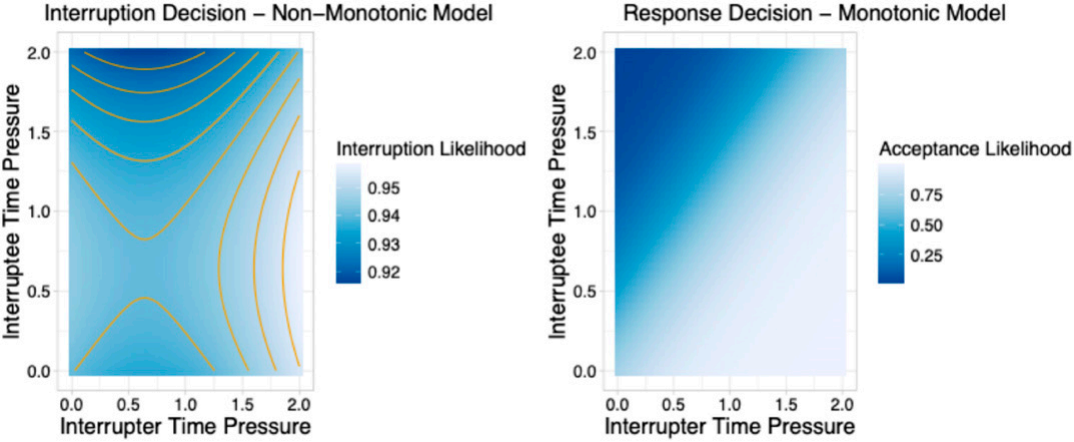
Predicted effect of interrupter and interruptee time pressure on interruption (left) and response (right) decisions according to the LOO-IC selected models. *Note.* Orange lines are included in the interruption decision model plot to highlight the non-monotonic pattern.

Figure 7 shows the predictions of the LOO-IC favored models. The non-monotonic interruption decision model (left panel of Figure 7) suggests that participants had a high likelihood of interrupting overall, as supported by the credible and positive parameter estimate for interrupter bias. Interrupter and interruptee time pressure had a weak non-monotonic influence on participants likelihood of interrupting. When interrupter time pressure was high and interruptee time pressure was low to medium, participants were most likely to interrupt, and when interrupter time pressure was low-medium and interruptee time pressure was high, participants were least likely to interrupt. However, in line with the non-credible interrupter sensitivity parameters, the effects of time pressure on interruption likelihood were weaker when time pressure was low-medium for both nurses. Thus, participants had a tendency toward interrupting, with the interruptee’s time pressure only reducing their likelihood of interrupting when it was very high.

When making response decisions, the hypothesized monotonic model suggests that participants were much more sensitive to any difference in time pressure between interrupter and interruptee (right panel of Figure 7). In line with the credible interruptee sensitivity parameter, the likelihood of accepting interruptions increased when the interrupter’s time pressure was greater than the interruptee’s time pressure and decreased when the interrupter’s time pressure was less than interruptee’s time pressure.

**Figure 8.**
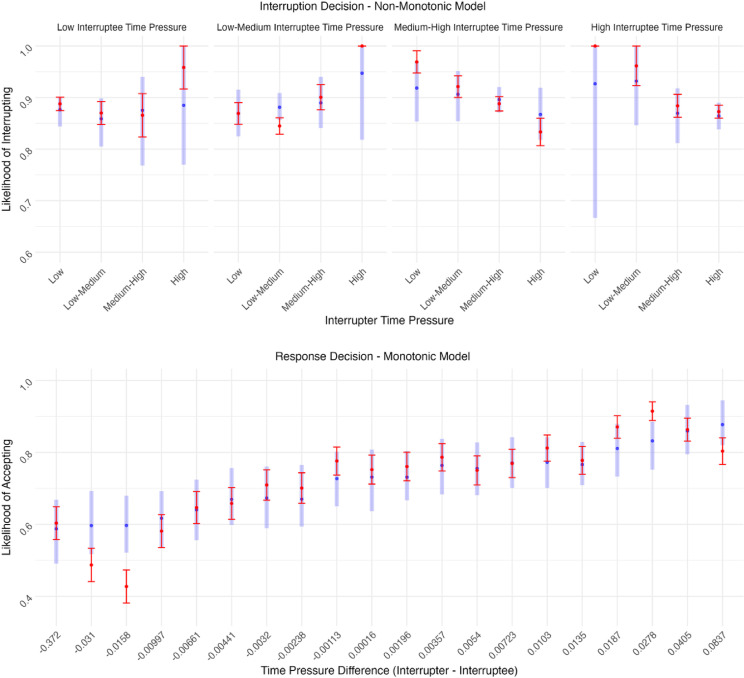
Posterior predictive distributions of the LOO-IC favored interruption decision and response decision models. *Note.* Thick blue lines represent model predictions from the best-fitting models according to LOO-IC, with the blue dot indicating the mean prediction. Thin red lines represent the observed data, including the mean (dot) and standard error bars.

Finally, the posterior predictive plots (Figure 8) depict how accurately the best-fitting model predictions represent observed decisions, by showing model predictions in blue and participant data in red. First, it is important to acknowledge that there was considerable variation between participants in their likelihood of interrupting and accepting interruptions, as reflected by the mean and standard errors bars in red. The top of Figure 8 shows that the non-monotonic model accurately accounts for participants interruption decisions, as the likelihood of interrupting changed in a non-monotonic way with interrupter (horizontal axis) and interruptee time pressure (panels), in line with observed decisions. When interruptee time pressure was low or low-medium, the likelihood of interrupting increased as interrupter time pressure increased, but when interruptee time pressure was medium-high or high the opposite was true—the likelihood of interrupting decreased as interrupter time pressure increased. Similarly, the hypothesized monotonic model (bottom of Figure 8) accurately accounts for participants response decisions, as, in line with observed decisions, the likelihood of accepting interruptions increased as the interrupter’s time pressure rose in comparison to the interruptee’s.

## Discussion

In this paper, we empirically tested a computational model of interruptions processes and effects in a simulated ICU. Participants acted as a nurse working on tasks to discharge two patients from an ICU while interacting with a second nurse through interruptions. We examined how progress and time remaining influenced the likelihood of interrupting and accepting interruptions. We also fit the interruption and response decision models to data to estimate parameters and compare the models with an alternative. In the following sections, we (1) summarize our findings and discuss contributions and implications, (2) discuss suggestions for future intervention design and further model testing, and (3) highlight considerations and avenues for future research.

### Study Contributions and Implications

Previous interruptions research has largely relied on verbal theories, with little consideration of how interruptions affect an entire team (Grundgeiger & Sanderson, 2009; Santomauro et al., 2021; Westbrook et al., 2010). There have been a limited number of computational models developed in the field, such as Altmann and Trafton (2002) Memory for Goals model which represents how interruptions and the associated resumption lags affect goal progress. However Knight et al.’s (2023) model is the first to examine interruption and response decision making in safety-critical environments from the perspective of both the interrupter and the interruptee. In this paper, we empirically tested the computational model to provide a more holistic understanding of how interruptions and responses occur within safety-critical environments.

Model fitting revealed that the processes underlying how participants made interruption decisions were different to those underlying how they made response decisions, with considerable variation between participants. On average, contrary to the hypothesized model, the decision to interrupt was not as straightforward as assessing the difference in time pressure between interrupter and interruptee (Ebright et al., 2003; Gao, 2018; Rivera, 2014). The best-fitting non-monotonic model suggests that participants were more likely to interrupt when their time pressure is much greater than the interruptee’s time pressure and would be least likely to interrupt when their time pressure is much less than the interruptee’s time pressure. However, when time pressure is not particularly high for the interrupter or interruptee, they would be generally biased toward interrupting.

Contrastingly, the response decision process occurred in line with the literature. Participants had a smaller bias toward accepting interruptions than they did toward interrupting. They weighed up the benefit to the interrupter and cost to themselves in a monotonic way and were more likely to accept interruptions when the interrupter was under more time pressure than they were (Colligan & Bass, 2012; Ebright et al., 2003). For example, results suggest that team members may be more inclined to help one another and handle interruptions if they can see that the interrupter is under more time pressure than they are, in line with research suggesting that nurses often work cooperatively (Ebright et al., 2003; St Onge & Parnell, 2015; Walter et al., 2014; Zheng et al., 2019).

Further, participants appeared sensitive to the likelihood of themselves and their coworker completing tasks on time when deciding whether to interrupt or accept interruptions. Results suggest that team members may be more likely to interrupt when it is imperative to their own task completion within the deadline (i.e., they have made good progress and have little time remaining), and less likely to interrupt when an interruption will not help their chances of successful task completion (i.e., poor progress and little time remaining). Results also suggest that team members would be more likely to interrupt when the interruptee is able to complete their tasks within the deadline, even if interrupted (i.e., the interruptee has made good progress and has plenty of time remaining), and when the interruptee is not going to complete tasks within the deadline, regardless of being interrupted (i.e., poor progress and little time remaining). Finally, team members would be more likely to accept interruptions when their chances of task completion were low, as they may recognize that they would not complete all tasks on time, regardless of the interruption.

The apparent sensitivity to the interrupter and interruptee’s chances of successful task completion extends beyond previous research which suggests that people would be more likely to interrupt or accept interruptions if the interrupter’s time pressure is greater than the interruptee’s (Ebright et al., 2003; Gao, 2018; Rivera, 2014). However, it is important to test the findings with participants who work in safety-critical environments. If such findings hold true with a nursing population for example, they will have important implications for practice. Although interrupting when the interruptee’s chances of completion are low would not be consequential for the interruptee’s task completion within the deadline, such a decision could increase the time pressure and risk of error for the interruptee further. Likewise, the interrupter choosing not to interrupt or accepting an interruption when their own chances of completion are low would not be consequential for their own task completion within the deadline, but could increase their time pressure and risk of error, thus posing a risk to safety (Gao, 2018; Johnson et al., 2017; Manias et al., 2021; Santomauro et al., 2021; Westbrook et al., 2010).

### Intervention Design and Future Model Testing

Previous interventions focused on the negative consequences of interruptions have not been widely successful (Gao et al., 2017). Our findings about how people make interruption and response decisions can help to guide future interventions aimed at reducing interruptions, in way that best suits the team.

Interventions could be designed to train safety-critical workers on interruption and response decision making. Participants had a strong tendency toward interrupting and were less likely to interrupt only when the interruptee’s time pressure was much greater than their own. However, when making response decisions, participants were sensitive to differences in time pressure and were more likely to accept interruptions as the interrupter’s time pressure increased in comparison to their own. Future interventions could focus on training workers to make interruption and response decisions that are consistent. When making interruption decisions, workers could be trained to conduct a fairer assessment of benefit and cost, even when the difference is small, to reduce the general tendency toward interrupting.

Workers could also be trained to assess the potential impact of post-interruption errors when deciding whether to interrupt or accept interruptions when the chances of task completion within the deadline for their team member or themselves are low. Although interrupting or accepting interruptions in these situations would not noticeably affect chances of successful task completion, it could pose a notably risk to safety, increase time pressure, and decrease team efficiency (Gao, 2018; Johnson et al., 2017; Manias et al., 2021; Santomauro et al., 2021; Westbrook et al., 2010).

While our findings provide initial guidance for interventions, modeling is an iterative process (Ballard et al., 2019; Kozlowski et al., 2013; Mäs et al., 2013; Weinhardt & Vancouver, 2012), and the models should continue to be refined to ensure that it accurately reflects human behavior before engaging it for intervention design. Specifically, findings could be tested with participants from safety-critical work environments, such as nurses, pilots, or air traffic controllers.

An advantage of the models and accompanying experiment is that they are openly available (https://osf.io/asgp7/) and highly flexible, so can be adapted to represent range of interruptions, tasks, team compositions, or settings. For example, the models and experiment could represent tasks such as medication administration in an emergency ward, where interruptions frequently occur, and where time pressure may be more salient (Chisholm et al., 2001; Westbrook et al., 2018). Different team compositions or patient allocations could also be represented, by having more nurses that could be interrupted, or having nurses care for a greater load of patients. The models and experiment can also be adapted to represent a different setting, such as a team working in an aircraft cockpit (Gontar et al., 2017; Latorella, 1998). Finally, models can generate other predictions, such as how the risk of error increases post-interruption, or the cumulative impact of interruptions and errors (Mark et al., 2008).

### Additional Considerations and Avenues for Future Research

Our experimental findings provide insight into how people make interruption and response decisions in a simulated safety-critical workplace. However, there are some limitations worth noting. First, when participants were required to respond to an interruption, the message overlayed their current task question. As such, participants may have already felt interrupted, so were more willing to accept the interruption. In future, interruptions could appear as a message bubble that does not obstruct the primary task. Future research could also incorporate different types of interruptions, including those that are not necessary for the task to be completed, or that occur more frequently or at unexpected times, as can happen in hospital wards (Khairat et al., 2021; Sasangohar et al., 2014; Schneider et al., 2021; Westbrook et al., 2017).

Second, time pressure was captured in the experiment by showing participants’ theirs and the simulated nurse’s deadline, their time used, and progress. However, to avoid missing data, we allowed participants to continue once the deadline had passed, which occurred in 100% of short, 70% of medium, and in 14% of long deadline condition blocks. This may have reduced participants sense of time pressure in the second block if they realized they could continue past the deadline. The experimental tasks could instead finish once the deadline is reached, with participants given a “team efficiency score.” Further, participants may have varying degrees of sensitivity to the approaching deadline. Tasks in safety-critical environments may not always have specified deadlines, so workers perceptions of time pressure may be more subjective. In future, participants could report their subjective rating of time pressure after each block.

Third, there was no way to verify the extent to which participants were attending to information such as progress and time used. In future, the experiment could be run with an eye-tracking device, to see what information participants primarily look to when making interruption and response decisions.

Fourth, in future experiments, researchers could manipulate different variables and test different outcomes using the openly available models and experiment. For example, researchers could manipulate and assess how the importance of tasks for maintaining productivity and safety, which was held constant in our experiment, might affect interruption and response decisions. In a nursing context, the importance variable could reflect patient acuity, capturing the importance of tasks for patient safety. The models also assume that all errors are detected, as is most often the case (Tzanetos et al., 2020; Zhao et al., 2017) and the consequence lies in the time taken to resolve them. However, if errors are left unresolved, particularly during medication tasks, they can lead to significant harm (Hall et al., 2010; Thomas et al., 2017; Westbrook et al., 2010). Researchers could therefore introduce a new outcome measure to assess how errors that are undetected accumulate over time and affect safety.

Fifth, by running the experiment with a human participant and simulated nurse, the simulated nurse’s behavior on tasks could be informed by the models and pilot testing, allowing greater experimental control. However, social factors, such as the demeanor of the interruptee (Rivera, 2014), were therefore not accounted for. Thus, future experiments could be conducted with two human participants to assess for any changes in the overall tendency toward interrupting or accepting interruptions.

Finally, we required a large sample for the experiment to conduct Bayesian hierarchical modeling (Knight, Neal, et al., 2023) and chose to recruit the more accessible general population, as this was the first study using the paradigm. However, it is worthwhile recruiting safety-critical workers, particularly nurses, in future research. Due to the time pressure under which workers in safety-critical environments often work (College of Intensive Care Medicine of Australia and New Zealand, 2011; Ebright et al., 2003; Lin et al., 2009), they may have different sensitivity to time pressure than the general population. For example, they may be more sensitive to time pressure at all levels when making interruption decisions, or they may place less emphasis on time pressure, giving more consideration to factors such as importance or patient acuity. Nurses may also complete experimental tasks faster and more accurately than the general population. However, the speed and accuracy of non-nursing participants in the current study is unlikely to have affected results as the deadlines (and therefore time pressure) were calibrated to match the speed of non-nursing pilot participants.

### Conclusion

In this study we developed an experimental paradigm that allowed for direct testing and comparison of computational models of interruptions. We ran a human-in-the-loop experiment with 312 participants and found that factors such as progress and time remaining influenced participants’ decisions to interrupt and accept interruptions. Through model fitting, we also found that participants considered the time pressure of the interrupter and interruptee in different ways. Findings provide insight into the differences underlying how people make interruption versus response decisions. The openly available models can continually be tested and used to inform future interventions to be designed in a way that is best suited for the entire team.

## Key Points

• Interruptions in safety-critical workplaces are associated with increases in errors, which can be detrimental for safety. Although interruptions can disrupt the interruptee, they may be necessary for the interrupter to maintain safety.
• We took a computational modeling approach to better understand the interruptions process from the perspective of both the interrupter and interruptee. Specifically, we tested a computational model that represents how the interrupter and interruptee make decisions to interrupt and respond to interruptions from one another, by considering factors such as the time pressure of tasks.
• To test the computational model, participants played the role of a nurse working in a team to discharge patients from an ICU to a general ward, while being required to make decisions to interrupt or respond to interruptions.
• Through Bayesian hierarchical modeling, we found that the non-monotonic model, rather than the monotonic model, provided the best explanation of the data for interruption decisions. Participants were biased toward interrupting, with time pressure only influencing their decisions when it was very high. Contrastingly, the monotonic model best explained response decisions. Participants were more likely to accept interruptions as the interrupter’s time pressure increased in comparison to their own.
• Results provide insight into the different processes underlying interruption and response decisions and may be used to inform future interventions, particularly to improve workers assessment of time pressure when making interruption decisions.
